# Supplementary material for: Enhanced antibiotic resistance development from fluoroquinolone persisters after a single exposure to antibiotic
Source: Nat Commun. 2019 Mar 12;10:1177. doi: 10.1038/s41467-019-09058-4 (PMC6414640; doi:10.1038/s41467-019-09058-4)
Supplement: Supplementary file 1 — Supplementary Information [file 41467_2019_9058_MOESM1_ESM.docx]

**Supplementary Information**

Enhanced antibiotic resistance development from fluoroquinolone persisters after a single exposure to antibiotic

Barrett *et al.*

**SUPPLEMENTARY METHODS**

**Bacterial strain construction**

Bacterial strains used in this work are indicated in Supplementary Table 2. The method of Datsenko and Wanner ^1^ was used to make MG1655Δ*recA* and Δ*umuDC*; *recA* and *umuDC* were replaced with a kanamycin resistance (KAN^R^) cassette amplified from pKD4, whereas *polB* and *dinB* were deleted by P1 transduction of deletions from the Keio collection ^2^. The KAN^R^ cassettes were subsequently removed using FLP recombinase expressed from pCP20 ^1^. The primers listed in Supplementary Table 3 were used to confirm mutants. To generate MG1655 *lexA3*, *malK* was first deleted by P1 transduction and clones were selected on LB agar with 50 μg/mL KAN. Deletion of *malK* produced a maltose auxotroph. *lexA3* was then transduced into MG1655Δ*malK*::KAN^R^, and transductants were selected on minimal media with maltose as the sole carbon source ^3^. Gibson assembly ^4^ was performed to generate pTB01 and pTB02 used for *recA* and *umuDC* complementation, respectively. The NEBuilder Assembly tool (New England Biolabs, Ipswich, MA) was used to design primers (shown in Supplementary Table 3) for pBAD33 ^5^, P*_recA_*-*recA*, and P*_umuDC_*-*umuDC* amplification, and the NEB Gibson Assembly Cloning Kit was used to assemble these vectors. To construct pTB01, the *recA* open reading frame (ORF) and 90 upstream nucleotides (nt) were amplified. For pTB02, the *umuDC* ORFs and 70 nt upstream of *umuD* were amplified. These upstream sequences encompass the transcription start sites of *recA* and *umuD* along with LexA binding sequences. The plasmid sequences were confirmed with PCR using the primers listed in Supplementary Table 3 and sequencing (Genewiz, South Plainfield, NJ).

**MIC test**

Strains were inoculated from -80 °C 25% glycerol stocks into test tubes containing 2 mL LB (containing 25 μg/mL chloramphenicol [CM] if plasmid retention was needed), and grown for 16 h at 37 °C with shaking (250 rpm). Samples were washed once with 0.85% NaCl, and diluted to OD_600_~0.2 in 0.85% NaCl. A sterile swab was used to inoculate the surface of a Mueller-Hinton (MH) agar plate (containing 25 μg/mL CM if plasmid retention was needed) of ~4 mm thickness by swiping from side-to-side across the entire surface of the plate three times, rotating the plate ~60° each time. Plates were dried at room temperature (~20 min), then an ofloxacin (OFL) Etest strip (bioMérieux, Marcy-l’Étoile, France) was placed on the plate. Plates were incubated at 37 °C for 20 h, at which time the MIC was read as the location where the ellipse of growth inhibition intersected the Etest. To ensure that the culture used to inoculate the plates was at a density of ~1-5 x 10^8^ CFU/mL, the culture was diluted further in 0.85% NaCl and plated to obtain single colony resolution on MH agar plates without Etests, and colonies were counted after incubation. Tests were performed three or more times on each strain. As depicted in Supplementary Fig. 1b, MICs were found to be 0.064 μg/mL OFL for wild-type MG1655, 0.008 μg/mL for MG1655Δ*recA*, 0.004-0.006 μg/mL for MG1655Δ*recA* bearing empty pBAD33, and 0.032-0.047 μg/mL for MG1655Δ*recA* bearing P*_recA_-recA*+pBAD33 (pTB01).

**Antibiotic persistence assay**

*E. coli* strains were inoculated from -80 °C 25 % glycerol stocks into test tubes containing 2 mL LB (containing 50 μg/mL KAN or 25 μg/mL CM if plasmid retention was needed), and grown for 5 h at 37 °C with shaking (250 rpm). Samples were centrifuged at 15k rpm for 3 minutes, supernatant was removed, and samples were resuspended in an equivalent volume of fresh M9 minimal media containing 10 mM glucose as the sole carbon source (M9-glucose). Samples were then inoculated into 25 mL M9-glucose in 250 mL baffled flasks to OD_600_~0.01. Cultures were grown for 20 h at 37 °C with shaking (250 rpm). When different treatment conditions were to be assayed, a common 5 h LB starter culture was used to inoculate separate flasks for the 20 h growth period. At the completion of 20 h of growth, a sample from each flask was removed, washed with PBS, serially diluted in PBS, and 10 μL of each dilution was plated on LB agar to enumerate CFUs/mL. Antibiotic-treated samples were treated with 5 μg/mL OFL or 1 μg/mL ciprofloxacin (CIPRO), while an equivalent volume of sterile deionized water was added to the untreated samples. Samples were incubated 5 h at 37 °C with shaking (250 rpm). At designated time points, 1 mL samples were removed and centrifuged for 3 min at 15k rpm. After removing the supernatant, pellets were resuspended in 1 mL PBS. This wash step reduced antibiotic concentrations ~50-fold. Wash steps were repeated at least once more to further dilute antibiotic concentrations another ~50-fold (~2,500-fold dilution from the two washes, such that ~0.002 μg/mL of OFL remains) in order to ensure that OFL concentrations were below each strain’s respective MIC (0.064μg/mL OFL for wild-type MG1655, 0.008μg/mL for MG1655 Δ*recA*, 0.004-0.006 μg/mL for MG1655Δ*recA* bearing empty pBAD33, and 0.032-0.047 μg/mL for MG1655Δ*recA* bearing P*_recA_-recA*). For Δ*recA,* Δ*recB,* Δ*ruvA,* Δ*recG,* and Δ*recG* Δ*ruvA*, samples were centrifuged for 3 min at 15k rpm after the final wash step, 800 μL supernatant was removed and the sample was resuspended in the remaining 200 μL of supernatant (5-fold concentration of samples to lower the limit of detection for these strains). All samples were serially diluted 5- or 10-fold in PBS, and 10 μL spots of each dilution were plated on LB agar. Plates were incubated for 16 h at 37 °C, and then CFUs were quantified from spots that typically contained 10-50 colonies.

When sequential persistence assays were performed, OFL persisters were washed to remove the antibiotic, cultured in LB, and stocked in 25% glycerol at -80 ^o^C. Those stocks were inoculated and cultured to stationary phase following the protocol described above. The cultures were treated with 5 μg/mL OFL (or equal volume of deionized water for the untreated control) for 5 h before OFL was removed and persisters were enumerated. In these assays, indistinguishable survival curves were obtained (Supplementary Fig. 1a). This type of assay has been used to demonstrate the phenotypic nature of persistence, as survival was not enhanced in persister-derived cultures ^6,7^.

**Quantification of resistance development from persistence**

To quantify the development of antibiotic resistance from OFL or CIPRO persisters, OFL or CIPRO persistence assays were performed following the methods described above. At the completion of OFL or CIPRO treatment, two 1 mL samples from each flask were removed, and antibiotic was removed by washing in PBS as described above (resulting in ~2,500-fold dilution of the antibiotic, such that ~0.002 μg/mL OFL remains). One of the aliquots was serially diluted in PBS and 10 μL of each dilution was spotted on LB agar to quantify cell survival. The second aliquot was centrifuged for 3 min at 15k rpm, and ~700 μL supernatant was removed leaving ~300 μL, in which the pellets were resuspended. The entire sample was then inoculated into 25 mL LB in a 250 mL flask or MOPS-EZ media (Teknova EZ Rich Defined media lacking glucose that was used for DNA quantification and its corresponding resistance assay), which further dilutes the OFL 84-fold for a combined ~210,000-fold dilution (~ 0.00002 μg/mL OFL remaining), and cultures were incubated at 37 °C with shaking (250 rpm). To enumerate resistant colonies derived from an untreated control, a culture that was treated with deionized water instead of OFL or CIPRO during the persistence assay was washed and recovered in LB or MOPS-EZ. After 8, 12, or 16 h, ~10^9^ cells were transferred to microcentrifuge tubes to quantify the number of resistant mutants. We note that we also attempted to quantify resistant colonies after 2 h and 4 h of recovery but were unable to obtain 10^9^ viable CFUs from one 25 mL culture. Samples were centrifuged for 3 min at 15k rpm, and supernatant was removed leaving ~100 μL. Pellets were resuspended in the remaining supernatant and plated on LB agar containing 175 ng/mL OFL, 500 μg/mL RIF, 30 μg/mL carbenicillin (CARB), 100 μg/mL D-cycloserine (DCS), or 20 μg/mL fosfomycin (FOS). Concentrations of antibiotics were selected so that ~10-100 resistant colonies were present for the untreated population. Another sample was serially diluted and plated on LB agar to determine total CFUs plated on antibiotic-containing agar; CFUs on these plates were counted after incubation at 37 ^o^C for 16 h. Resistant colonies were enumerated after incubation at 37 ^o^C for 24 h. We note that we observed pinpoint colonies on DCS and FOS plates following overnight incubation (for both the untreated and OFL persister-derived populations). However, only larger colonies were able to grow and form colonies when they were struck out on fresh plates containing 100 μg/mL DCS or 20 μg/mL FOS, whereas the pinpoint colonies failed to do so. Thus, pinpoint colonies were excluded from resistant colony enumeration.

To determine the impact of the trace amount of OFL that would remain following washing in PBS and inoculation in LB (~210,000-fold dilution resulting in 0.02 ng/mL of OFL) on RIF resistance development, we inoculated 1 mL of the untreated population in LB with 0.02 ng/mL of OFL following washes in PBS. After 16 h of growth, ~10^9^ CFUs were plated on LB agar with 500 μg/mL RIF. Plates were incubated at 37 ^o^C for 24 h before colonies were enumerated. Fold change in resistant colonies in this population and in untreated population grown in LB without OFL was calculated and shown in Supplementary Fig. 6b. For all resistance development experiments, data were obtained as paired samples that were processed side-by-side (at the same time with identical media, identical antibiotic-containing plates, etc.). This controlled for any batch-to-batch differences in LB, which is a poorly-defined media. Raw data from these experiments are provided in Supplementary Fig. 6a and 7. We statistically analyze fold-changes of paired samples (*e.g.*, OFL-treated/untreated), which is a metric that controls for sources of experimental variance.

**Isolation of pre-existing antibiotic-resistant cells in stationary-phase populations prior to treatment**

For isolation of antibiotic-resistant cells present in stationary-phase populations prior to treatment, the growth conditions outlined above for antibiotic persistence assays were followed. After culturing cells in M9-glucose for 20 h, ~10^9^ CFUS were plated on LB agar plates containing 175 ng/mL OFL, 500 μg/mL RIF, 30 μg/mL CARB, 100 μg/mL DCS, or 20 μg/mL FOS. Plates were incubated 24 h at 37 °C. Following incubation, all CFUs were harvested from the plate with LB and centrifuged for 3 min at 15k rpm, supernatant was removed, and the pellet was resuspended in 1 mL fresh LB. The sample was incubated ~5 h at 37°C with shaking, then stocked in 25% glycerol. OFL persistence assays with these pre-existing resistant mutants were then performed following the procedure outlined above. Pre-existing OFL^R^ and DCS^R^ mutants had significantly higher tolerance levels than wild-type (Fig. 3b). If it is assumed that resistant mutants and wild-type have comparable growth-rates during the recovery period and that resistant mutants do not revert to antibiotic sensitive cells with an appreciable rate, the following expressions describe how pre-existing resistant mutants would impact the fold changes in resistant mutants between OFL persister-derived populations and untreated populations.

$FC=\frac{N_{\text{R}}^{\text{t}}}{N_{\text{R}}^{\text{u}}}=\frac{\text{Number of resistant mutants from OFL-treated culture}}{\text{Number of resistant mutants from untreated culture}}$ (1)

$N_{\text{R}}^{\text{i}}=N_{\text{R0}}^{\text{i}}E^{\text{i}}+N_{\text{RN}}^{\text{i}}=N_{\text{R0}}^{\text{i}}E^{\text{i}}+N_{\text{P0}}^{\text{i}}(E^{\text{i}}-1)f_{\text{R}}$ (2)

$N_{\text{P0}}^{\text{t}}=N_{\text{P0}}^{\text{u}}f_{\text{P}}$ (3)

$N_{\text{R0}}^{\text{t}}=N_{\text{R0}}^{\text{u}}f_{\text{PR}}$ (4)

$E^{\text{t}}=\frac{E^{\text{u}}}{f_{\text{P}}}$ (5)

$FC=\frac{N_{\text{R}}^{\text{t}}}{N_{\text{R}}^{\text{u}}}=\frac{N_{\text{R0}}^{\text{u}}E^{\text{u}}\left(\frac{f_{\text{PR}}}{f_{\text{P}}}\right)+N_{\text{P0}}^{\text{u}}\left(E^{\text{u}}-f_{\text{P}}\right)f_{\text{R}}}{N_{\text{R0}}^{\text{u}}E^{\text{u}}+N_{\text{P0}}^{\text{u}}\left(E^{\text{u}}-1\right)f_{\text{R}}}$ (6)

$$\text{i}=\text{ denotes OFL-treated (t) or untreated (u) }$$

$$N_{\text{R0}}^{\text{i}}=\text{ number of resistant mutants at time 0 of recovery in culture i}$$

$E^{\text{i}}=\text{ expanision of culture i during recovery (final number of cells divided by initial number of cells)}$ $N_{\text{RN}}^{\text{i}}=\text{ number of resistant mutants generated by de novo mutation during recovery in culture i}$

$$N_{\text{P0}}^{\text{i}}=\text{ number of non-resistant cells at time 0 of recovery in culture i }$$

$$f_{\text{R}}=\text{ frequency of de novo mutation to resistant mutant}$$

$$f_{\text{P}}=\text{ fraction of stationary-phase wild-type cells that are OFL persisters }$$

$$f_{\text{PR}}=\text{ fraction of stationary-phase resisant mutant cells that are OFL persisters }$$

If pre-existing resistant mutants dominate the abundance of resistant mutants after recovery, the expression for FC simplifies to:

$FC=\frac{N_{\text{R}}^{\text{t}}}{N_{\text{R}}^{\text{u}}}=\frac{N_{\text{R0}}^{\text{u}}E^{\text{u}}\left(\frac{f_{\text{PR}}}{f_{\text{P}}}\right)}{N_{\text{R0}}^{\text{u}}E^{\text{u}}}=\frac{f_{\text{PR}}}{f_{\text{P}}}$ (7)

For DCS, pre-existing mutants are 4.5-fold more tolerant than wild-type (*f_PR_*/*f_P_* = 4.5), so the fold-change for DCS experiments should yield a value of 4.5 if enrichment of pre-existing mutants was the only factor contributing to the higher DCS^R^ mutant levels in populations derived from OFL persisters. The enhancement in resistance for DCS was 20-fold, which was significantly larger than 4.5-fold. For OFL, pre-existing mutants are 19.5-fold more tolerant, and the enhancement in resistance was not significantly different from that value.

###

### **Determination if recovery is essential for enhancement of RIF resistance in OFL persister-derived populations**

In order to determine if recovery was essential for enhancement of RIF resistance in OFL persister-derived populations, an antibiotic persistence assay was performed following the procedures described above, and cultures were plated on RIF-containing LB agar just prior to the assay and at its conclusion. Before treatment with OFL (“before treatment” in Fig. 3c), approximately 10^9^ CFUs were plated on LB agar containing 500 μg/mL RIF. To assess whether post-treatment recovery in LB was needed for resistance enhancement, 10 mL of culture was washed following the steps described above to remove OFL after 5 h of treatment. These cells were then plated across five RIF-containing LB agar plates, so that we could plate ~10^9^ cells without accumulating excessive dead cells on each plate, and these plates were incubated at 37^o^C for 24 h to enumerate resistant CFUs (“after treatment without recovery” in Fig. 3c). Serial dilutions on antibiotic-free LB agar were also plated at each time point to quantify CFUs.

**Impact of reduced inoculum at recovery on RIF resistance**

To understand how the number of RIF resistant mutants observed after 16 h of recovery is influenced by the number of generations of growth for each population, we first conducted antibiotic persistence assays as detailed above. At the completion of OFL treatments and washing to remove the drug, samples were inoculated into 25 mL LB in a 250 mL flask for recovery at 37 °C with shaking (250 rpm) for 16 h as described above. At 0 h of recovery, 300 μL of each of the untreated and OFL persister-derived populations was removed to measure the OD_600_, and 1 mL was removed to quantify CFUs. This was repeated at 2 h, 4 h, 8 h, and 16 h of recovery. We note that at early time points of recovery for the OFL-treated sample, OD_600_ increased without a corresponding increase in CFUs due to filamentation of the cells in response to the preceding OFL treatment. To account for the lower abundance of culturable cells in the OFL-treated sample at 0 h of recovery, we washed 1 mL of the untreated sample as described above, resuspended the cells in 1 mL of PBS, and inoculated 20 μL of the this suspension into 25 mL LB in a 250 mL flask (50-fold reduced inoculum of the untreated sample, which approximated the number of culturable cells in the OFL-treated sample). As the number of CFUs in the OFL-treated sample decreased at 2 h, we performed additional experiments where the inoculum of untreated sample was reduced 100-fold by inoculating 10 μL of the washed and resuspended sample in 25 mL of LB. The OD_600_ and CFU/mL of the 50- and 100-fold reduced samples at inoculum are shown on Supplementary Fig. 6c and d. After 16 h of growth in LB for the 50- and 100-fold reduced samples, ~10^9^ cells were plated on LB agar containing 500 μg/mL RIF. At the same time, ~10^9^ cells from the untreated population and OFL persister-derived population were also plated on antibiotic-containing plates after 16 h of growth in LB. Resistant CFUs were enumerated after 24 h, and fold-change in resistant mutants arising from untreated samples (with and without inocula reduction), as well as from OFL persister-derived samples and untreated samples (with and without inocula reduction) were calculated and these data are shown in Supplementary Fig. 6e.

**DNA quantification to measure DNA amplification during recovery.**

Additional rounds of DNA replication could increase the likelihood of a mutation appearing. Thus, we quantified DNA abundance at the beginning and end of the 16 h recovery period using PicoGreen to compare the amount of DNA synthesized in the untreated and OFL-treated population during this time. To do this, we conducted persistence assays as described above. At the completion of treatment, two 1 mL samples from each flask were removed, and antibiotic was removed by washing in PBS as described above. One of the aliquots was serially diluted in PBS and 10 μL of each dilution was spotted on LB agar to quantify cell survival. The second aliquot was centrifuged for 3 min at 15k rpm, and ~700 μL supernatant was removed leaving ~300 μL, in which the pellets were resuspended. The entire sample was then inoculated into 25 mL MOPS-EZ media in a 250 mL flask, and cultures were incubated at 37 °C with shaking (250 rpm) for 16 h. We used MOPS-EZ media for this experiment as high green fluorescent background signals were observed with LB. At 0 h and 16 h of recovery, 1 mL of each of the OFL-treated population and the untreated control were transferred to microcentrifuge tubes, and the cells were lysed by sonication at 10% amplitude using a Fisher Scientific Model 50 Sonic Dismembrator on ice for 30 min. The lysates were diluted 10-fold (for the t=0 h sample) or 100-fold (for the t=16 h sample) in MOPS-EZ media and DNA was quantified using PicoGreen (Thermo Fisher Scientific, Waltham, MA). PicoGreen stock was dissolved in DMSO and diluted 1:1000 in MOPS-EZ, and 100 μL of diluted PicoGreen was incubated with 100 μL of diluted cell lysates in a black, clear-bottom 96-well plate (Corning Inc., Corning, NY) at room temperature for 5 min. Following the incubation, fluorescence was measured using a Synergy H1 Hybrid multimode microplate reader (BioTek, Winooski, VT) at excitation and emission wavelengths of 490 nm and 520 nm, respectively. To generate the standard curves used for DNA quantification, 10 mg/mL of UltraPure Salmon Sperm DNA Solution (ThermoFisher) was serially diluted to 0.2 to 2,000 ng/mL in MOPS-EZ (five dilutions). 100 μL of each diluted standard was incubated with 100 μL of diluted PicoGreen at room temperature for 5 min before fluorescence measurements. To assess whether the DNA that could have been excreted into the extracellular milieu due to cell death and lysis remained stable over the 16 h recovery period, lysates collected at t=0 h were filtered using a 0.22 μm filter to remove any unlysed cells and the filtrates were incubated at 37 ^o^C for 16 h. Immediately after filtration and after the 16 h incubation period, the samples were subjected to DNA quantification as described above, and we did not observe a decrease in DNA (Supplementary Fig. 6g).

**Time-lapse microscopy acquisition and growth and fluorescence quantification**

For time-lapse microscopy, LB pads with 1% agarose and 50 μg/mL KAN (included for reporter plasmid retention) were made. We used a preparation method that is modified from the one described by Young and colleagues ^8^. Microscopy slides, cover slips, and spatulas used for LB pads preparation were autoclaved at 121^o^C for 30 min to achieve sterilization. To prepare LB pads, Certified Molecular Biology agarose (Bio-Rad, Hercules, CA) was dissolved in LB using a microwave (Haier America, New York, NY) set to power level 4. LB-agarose was microwaved for ~2.5 minutes, until the liquid was boiling. The media was allowed to cool slightly, then antibiotic (50 μg/mL KAN) was added for plasmid retention. Approximately 1 mL of LB-agarose was spotted onto a 10.5 x 35 mm rectangular coverslip (Chemglass Life Sciences, Vineland, NJ), and another coverslip was placed on top. To control the thickness of the agarose pads, two microscopy slides were placed on either side of the coverslip before the liquid was pipetted and the second coverslip was placed on top. LB-agarose (with KAN) was allowed to harden ~1 h at room temperature, at which time the top coverslip was removed, and the LB-agarose was cut into four equally-sized pads using a sterile spatula.

After completion of the OFL persistence assay and washing as described above, samples were resuspended in 1 mL PBS, then diluted 1:12 in spent medium (media obtained from sterile filtering of the untreated sample). One μL of this diluted sample was spotted onto LB-agarose (with KAN) pads. A Lab-Tek chambered #1.5 coverglass (Thermo Fisher Scientific, Waltham, MA) was then placed on top of the pads so that the cells would be visible through the chambered coverglass. The entire system (chambered coverglass, pads, and rectangular coverslip) was inverted. The free edges of the rectangular coverslip were sealed with valap (1:1:1[w/w/w] of petroleum jelly, lanolin, and paraffin). As a control to assess sterility of the pads, an inoculum-free pad was always included in the chamber and imaged at the completion of the time-lapse experiment.

The microscope used was a fully-motorized Nikon Ti-E with Perfect Focus System equipped with Yokogawa spinning disc (CSU-21) mounted with a quad dichroic accommodating 405, 488, 561, and 647 lasers. The microscope was controlled using NIS-Elements, V4.5 (Nikon Instruments, Melville, NY). The spinning disc detector was an Orca Flash. An Agilent laser launch with a 488 laser was used for fluorescence imaging. The system was also equipped with a piezo stage with 100 μm range capable up to 100 steps/second. The objective used was CF160 Plan Fluor Phase Contrast DLL 100x oil objective with 1.3NA. An environmental control chamber mounted in a fully-motorized XY stage was used to keep the samples at 37 °C during the course of the experiment. Images were taken every 12 minutes for 16 h (for one replicate, images were obtained for only 10 h). At each time point, a 2-3 μm z-stack was taken in increments of 0.3 μm, a 2x2 tile at each XY position was taken, and both phase contrast and GFP fluorescence images were obtained. 300 ms exposure was used for both lasers, and 50% laser power was used for fluorescence imaging. At the completion of the time-lapse imaging, several images of each of the pads in areas not exposed to laser were taken to determine if qualitative difference between the cells exposed to and not exposed to laser were visible. No difference was seen for any experiment. Additionally, the inoculum-free pad was imaged to ensure sterility. The z-position with the sharpest contrast was chosen for each experiment, and tiles surrounding each XY point were stitched using the Stitching plugin ^9^ of Fiji ^10,11^.

MicrobeJ ^12^ was used to quantify growth and fluorescence of cells in time-lapse images. Default settings were used, with the exception of the following: Length: 1.1-max; Circularity: 0-0.89. Contours of cells were manually adjusted prior to calculation of results to ensure accuracy of particle detection. Fluorescence intensity presented is the corrected mean (mean_c) intensity value (average intensity value inside particle minus the average mean background intensity value). Persisters were quantified until the time point at which they divided. Non-persister ofloxacin-treated cells were quantified until the time point at which a portion of the cell exited the field of view or it lost its opacity, fluorescence, and stopped growing, or to the time point to which the persister in the image was quantified. Untreated cells were quantified until division or until crowding of cells hindered analysis by the software.

**Effect of trace levels of OFL on recovery observed with microscopy.**

Removing OFL from the treated samples by washing in PBS and preparing the samples for microscopy resulted in a ~30,000-fold dilution (~ 0.2 ng/mL OFL remaining). To determine the impact of this trace amount of OFL on the recovery period observed with microscopy, we diluted untreated cells 1:12 in spent media and plated 1 μL of this diluted sample on LB agarose containing 0.2 ng/mL OFL in preparation for microscopy, which was performed according to the above description.

**Assessing DNA damage with DAPI staining**

*E. coli* MG1655 cultures were grown overnight and treated with OFL for 5 h as described for persistence assays. As controls, untreated populations were also prepared. Following treatment, cells were washed and recovered in LB as described for persistence assays. At 0, 2, and 4 h of recovery, 1 mL of each culture was collected in microcentrifuge tubes and cells were pelleted by centrifugation at 15K rpm for 3 min. Pellets were then fixed with 1 mL of 4% paraformaldehyde (PFA) at room temperature for 30 min. After fixation, cells were pelleted by centrifugation and pellets were resuspended in PBS. As additional controls, *E. coli* MG1655 was inoculated into 2 mL of LB and grown for 16 h at 37 ^o^C, with shaking at 250 rpm. Following overnight growth, cells were diluted to OD_600_ ~0.01 in 25 mL of LB in three 250 mL baffled flasks and cultured to exponential phase (OD_600_ ~0.2-0.4). Cells were then treated with water (untreated control), 5 μg/mL of mitomycin (MMC; another drug that causes DNA breaks), or 10 μg/mL of piperacillin (PIP; inhibits penicillin binding proteins leading to filamentation without DNA damage) for 1 h. Cells were then fixed with PFA as described above. Samples were stored at 4 ^o^C until DAPI staining and imaging.

For DAPI staining, 100 μL of each sample, adjusted to OD_600_ ~0.2-0.4 in PBS, were pelleted by centrifugation. A 10 mg/mL stock of DAPI dissolved in DMSO was diluted 1,000-fold in PBS. Each pellet was resuspended in 100 μL of diluted DAPI and incubated in the dark at room temperature for 15 min. Stained samples were then pelleted by centrifugation, and pellets were resuspended in 100 μL of PBS. One microliter of each sample was spotted onto plain microscopy slides (Fisher Scientific) that were coated with of 0.1% (w/v) poly-L-lysine solution (Sigma-Aldrich). Before they were coated, slides were cleaned with 200-proof ethanol and 40 µL of the poly-L-lysine solution was added to the center of each slides. After 15 min at room temperature, the slides were rinsed three times with 1 mL of PBS and allowed to air dry. After each sample that was spotted onto the slide had dried, 5 μL of VectaShield fluorescence mounting medium (Vector Laboratory Inc., Burlingame, CA) was added to each slide before the cover glass was overlaid and sealed with clear nail polish. Cells were imaged using a Nikon TE2000 inverted microscope (Nikon, Melville, NY) equipped with a 100X Plan Fluor Nikon objective (1.45 NA). DAPI fluorescence was collected with a Nikon UV HYQ filter cube. A Hamamatsu Orca Flash 2.8 camera (Hamamatsu, Bridgewater, NJ) and NIS Elements software (Nikon) were used for image acquisition. ImageJ ^10^ was used for image analysis.

**DNA isolation for measurement of whole-genome mutation frequency**

In order to determine the mutation frequency in colonies derived from persisters and untreated cells, two cultures of wild-type MG1655 were grown to stationary phase, according to the persistence assay growth conditions detailed above. At the completion of 20 h growth, 1 mL samples were removed from each flask and genomic DNA was isolated using a Qiagen DNeasy Blood and Tissue Kit (Qiagen Inc., Germantown, MD). These samples provided the parental culture genomic DNA for whole-genome sequencing. Cultures were treated with 5 μg/mL OFL or equivalent volume of water (untreated control) for 5 h. Following treatment, a 1 mL sample was removed from each flask and centrifuged at 15 k rpm for 3 min, supernatant was removed, and the sample was resuspended in 1 mL PBS. These washing steps were repeated at least once more to ensure that the final OFL concentration was below the MIC (0.064 μg/mL OFL) for MG1655 (Supplementary Fig. 1b). Samples were plated on LB agar at a density that would provide single-colony resolution, and incubated at 37 °C for 16 h. Five individual colonies were then picked from each treatment condition. This experiment was performed twice, yielding 4 parental cultures, 10 persisters, and 10 untreated control samples. Genomic DNA was isolated from the individual colonies for whole-genome sequencing.

**Whole-genome sequencing (WGS) and data analysis**

WGS libraries were prepared using the Nextera DNA library preparation kit (Illumina, San Diego, CA). Samples from parental cultures, persisters, and untreated controls were sequenced in a 67 nt lane on the Illumina HiSeq 2500 sequencer in Rapid mode following the standard protocol (HiSeq Rapid SBS Kitv2), while samples from RIF-resistant colonies were sequenced as 30% of single-end 150 nucleotide lane on the same instrument. Raw sequencing reads were filtered by the Illumina HiSeq control software. Galaxy ^13-15^ was used to split reads by barcode, and Burrows-Wheeler Alignment (BWA) tool ^16,17^ was used to map reads to the reference *E. coli* chromosome (NCBI NC_000913.3). Nucleotides with a total read depth >10 were included in analyses. FreeBayes ^18^ was used to identify variants from reference nucleotides using the default settings, with the exception of a ploidy of 1. Galaxy was used to filter variants to those with an alternate allele frequency ≥0.5 (*i.e*., 50% or more of the reads at a specific nucleotide read a nucleotide that was not wild-type).

For the parental cultures, persisters, and untreated controls samples, Galaxy was used to downsample the number of mapped reads to ~5 million for each sample. This was done because there were variations in the total number of reads for these samples, and downsampling to an equivalent number of reads would ensure that variations in mutation frequencies between samples were not a result of variations in the total number of mapped reads. Because of the possibility for genetic heterogeneity in these samples, we chose not to set an arbitrary cut-off for calling a mutation (*e.g.* alternative nucleotide must be observed in over half the reads to be considered a mutation). Therefore, mpileup (SAMtools) was used to calculate read depth at each nucleotide and identify variants from reference nucleotides. Reference observation frequencies were calculated by dividing the number of times the reference nucleotide was read by the total read depth at that nucleotide.

Matlab (Mathworks, Inc.) was used to create dot plots of the reference allele frequency at each nucleotide in the parents versus their children (colonies derived from untreated cells or OFL persisters) using reference allele frequencies from mpileup data. Nucleotides that were not read in both the parent and children were excluded from dot plots. Additionally, because of the format of the mpileup output, which creates a second index for a nucleotide if an insertion is detected, thus prohibiting accurate calculation of the reference allele frequency at that nucleotide in a high-throughput manner, nucleotides at which insertions were detected were also not included in dot plots.

Matlab was used to bin reference allele frequencies into frequencies ≤0.25, ≤0.5, and ≤1. For this analysis, nucleotides with insertions and deletions were also eliminated since an accurate reference allele frequency at that nucleotide could not be calculated in a high-throughput manner.

**PicoGreen dsDNA quantitation assay to determine single cell chromosomal content**

A modified protocol from those of Akerlund and colleagues ^19^ and Ferullo and colleagues ^20^ was used to determine chromosomal content in single cells. At desired time points, OD_600_ of cultures were measured, and 1 mL of cultures were removed and permeabilized with 9 mL of 70% ethanol at 4˚C for three or more hours. Samples were then centrifuged at 4k rpm for 10 min and their supernatants were removed leaving ~1 mL, in which the pellets were resuspended and transferred to microcentrifuge tubes. Samples were centrifuged at 15k rpm for 3 min, and all supernatant was removed. To allow remaining ethanol to evaporate, samples were left with caps open (loosely covered with a low-lint tissue) overnight at room temperature. Samples were then resuspended in 1 mL PBS, and diluted to OD_600_~0.4 in 500 μL PBS. 100 μL of 1:100 dilution of PicoGreen in 25% DMSO was mixed with each sample, and samples were incubated for 3 h at room temperature in the dark. At completion of 3 h of incubation, 1 mL of 1:1000 dilution of PicoGreen (10-fold further dilution of 1:100 dilution, diluted in PBS) was added to each sample. Unstained controls were treated identically, except that they received equivalent volumes of diluents in place of PicoGreen. A LSRII flow cytometer (BD Biosciences, San Jose, CA) was used to acquire fluorescence intensities. Gating strategy is shown in Supplementary Fig. 8d-g. Forward and side scatter parameters (FSC and SSC) were used to identify microorganisms. A laser emitting at 488 nm was used for excitation of PicoGreen, and fluorescence intensities were collected with a 525/50 nm band-pass filter.

FlowJo software (TreeStar, Ashland, OR) was used to analyze samples. A PicoGreen-negative gate which captured 100% of the unstained populations was applied to stained populations to determine the delineation between PicoGreen-negative and PicoGreen-positive populations in the stained sample. Greater than 90% of the stained samples fell into the PicoGreen-positive gate for each of the three replicates.

A protocol adapted from Akerlund and colleagues ^19^ was used to determine the PicoGreen fluorescence intensities of subpopulations with 1, 2, 3, and 4 chromosomes. MG1655 *E. coli* was inoculated from a -80˚C stock into 2 mL LB and incubated with shaking (250 rpm) at 37˚C for 16 h. Cultures were then diluted 10^7^-fold into 25 mL LB containing 0.2% glucose in 250 mL baffled flask and covered with Breathe-Easy film (USA Scientific, Ocala, FL). Cultures were incubated at 37˚C with shaking (250 rpm), and samples were removed at 24, 48, 72, and 144 h. PicoGreen staining and analysis were performed as described above. With continued length of time in stationary phase, chromosomal content shifts towards 1 chromosome, as indicated by the peak at 50K in Supplementary Fig. 8i. Since PicoGreen intensity increases linearly with increasing dsDNA content^20^, chromosomal content can be inferred from the peaks corresponding to multiples of the 1 chromosome peak (*i.e.*, ~100K indicates 2 chromosomes, ~150K indicates 3 chromosomes, and ~200K indicates 4 chromosomes). Our data suggests that a small fraction of cells harbored three chromosomes, which is a ploidy that has been observed previously ^19, 21^.

**SUPPLEMENTARY FIGURES AND LEGENDS**


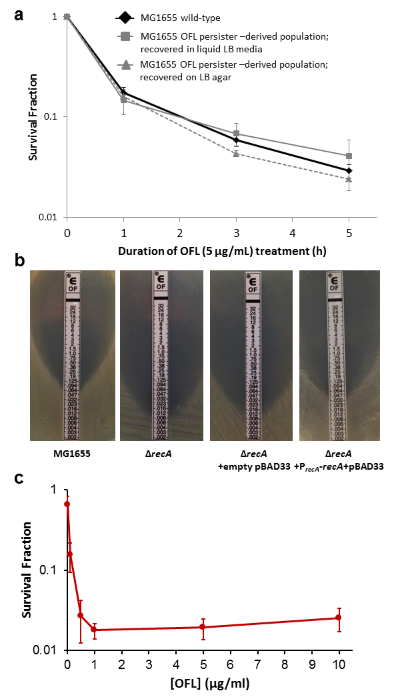


**Supplementary Fig. 1. OFL tolerance assays and MIC tests. (a)** MG1655 demonstrates biphasic killing with OFL under our experimental conditions (solid black line). OFL tolerance assays were performed on populations derived from persisters recovered in antibiotic-free liquid LB media (solid gray line) or antibiotic-free LB agar (dashed gray line). Progeny of persisters also demonstrate biphasic killing with OFL (n = 19 for wild-type; n = 4 for each OFL persister- derived population). Error bars portray S.E.M. **(b)** OFL Etests (bioMérieux, Marcy-l’Étoile, France) were used to quantify MICs of strains. MIC tests were performed three or more times on each strain. Representative images are presented. MICs were found to be 0.064 μg/mL OFL for *E. coli* MG1655, 0.008 μg/mL for MG1655 Δ*recA*, 0.004-0.006 μg/mL for MG1655 Δ*recA* bearing empty pBAD33, and 0.032-0.047 μg/mL for MG1655 Δ*recA* bearing P*_recA_-recA* in pBAD33 (pTB01). **(c)** When populations of *E. coli* MG1655 are treated with doses of OFL above 16-fold the MIC (≥1 μg/mL), the associated persister levels do not vary appreciably with increasing concentrations (n = 5).


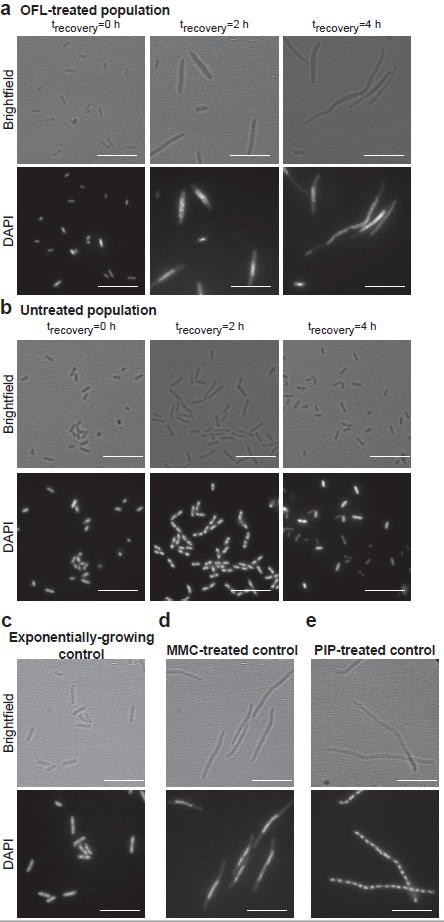


**Supplementary Fig. 2. DAPI staining of nucleoids.** OFL-treated cells **(a)** and untreated controls **(b)** were harvested and fixed immediately after inoculation in LB following treatment and after 2h and 4 h of recovery. Untreated cells resemble an exponentially-growing control **(c)**. At 4 h, most OFL-treated cells were filamentous and contain granulated nucleoids at mid-cell, which is characteristic of cells treated with DNA damaging agents, such as MMC **(d)**. Cells that have filamented because of treatment with piperacillin **(e)**, which is not a DNA damaging agent, do not share the same nucleoid physiology as OFL- and MMC-treated cells, but rather resemble untreated cells with a larger number of nucleoids. Images shown are representative of two (for panels **c**, **d**, and **e**) or three (for panels **a** and **b**) biological replicates. The scale bars represent 10 μm.


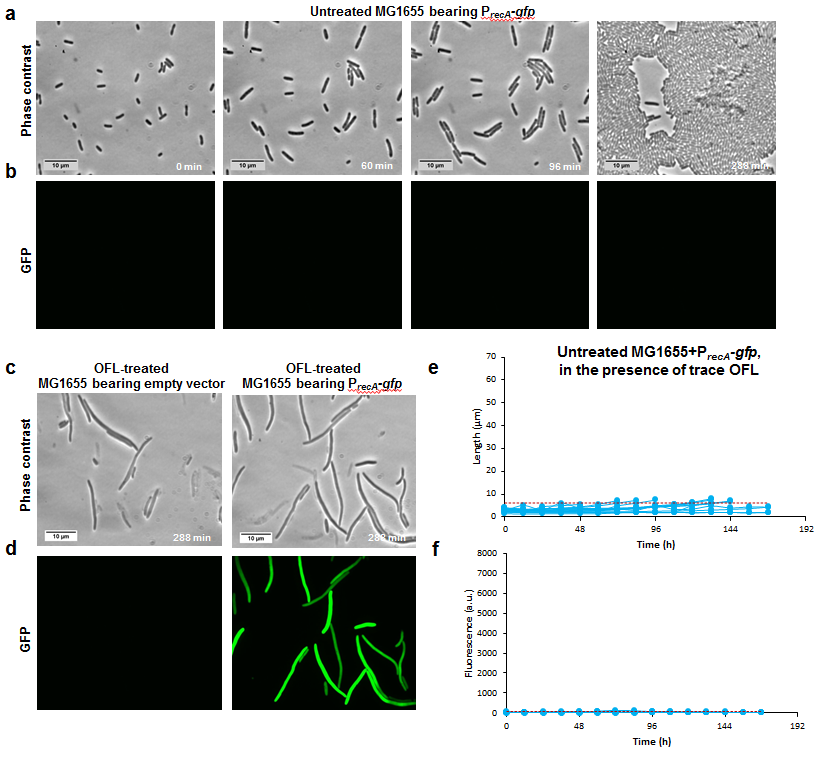


**Supplementary Fig. 3. Experimental Controls. (a)** Representative phase contrast and **(b)** GFP fluorescence images of recovering untreated wild-type *E. coli* MG1655 harboring the P*_recA_-gfp* reporter plasmid. Images are representative of six biological replicates. Refer to Supplementary Movie 3 for video. **(c)** Representative phase contrast and **(d)** GFP fluorescence images of recovering OFL-treated MG1655 with a promoterless reporter plasmid (left panel; n=2). OFL-treated MG1655 bearing P*_recA_-gfp* is shown for comparison (right panel). **(e-f)** Time-lapse microscopy was performed with untreated wild-type *E. coli* MG1655 bearing P*_recA_*-*gfp* reporter plasmid recovering in the presence of 0.2 ng/mL OFL (refer to Supplementary Movie 4 for video). **(e)** Growth and **(f)** fluorescence of 15 cells from two biological replicates were quantified, and 95% of cell length and fluorescence of the population lie below the red dashed line.


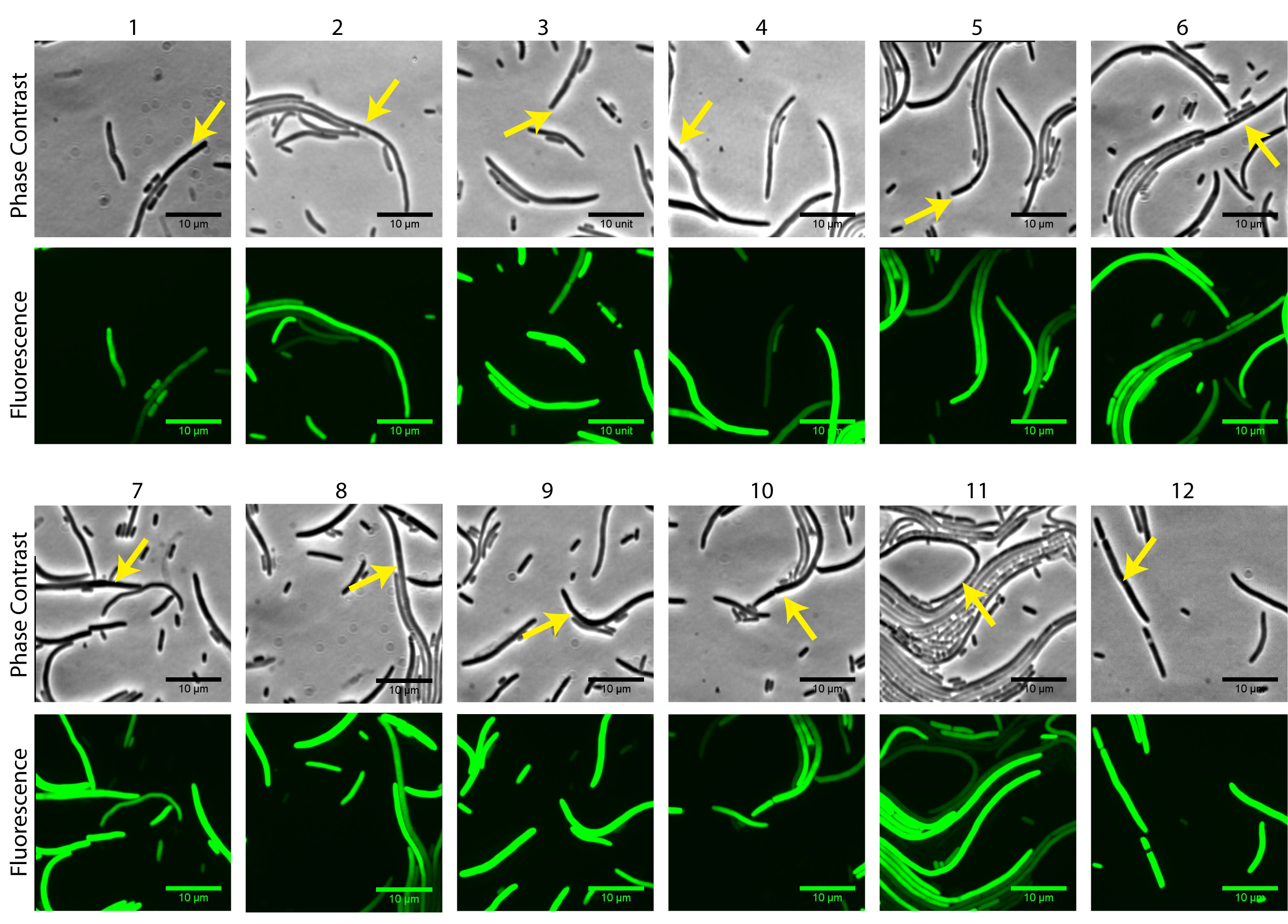


**Supplementary Fig. 4.** Phase contrast and fluorescence images depicting the 12 persisters that did not remain in the field of view throughout the course of the time-lapse imaging (indicated by yellow arrows) captured near the time that a division of the cell was observed. These cells were identified as persisters, as they continued to grow and divide after the time point shown in these images, eventually giving rise to microcolonies.


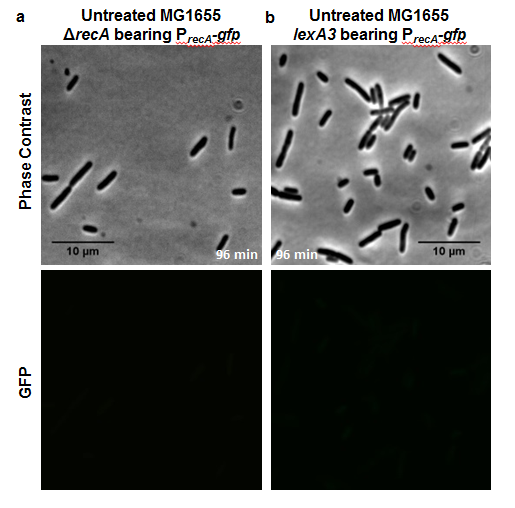


**Supplementary Fig. 5.** Representative phase contrast and GFP fluorescence images of recovering untreated **(a)** *E. coli* MG1655 Δ*recA* and **(b)** *lexA3* mutants harboring the P*_recA_-gfp* reporter plasmid. Images are representative of two biological replicates. Refer to Supplementary Movies 5-8 for video.


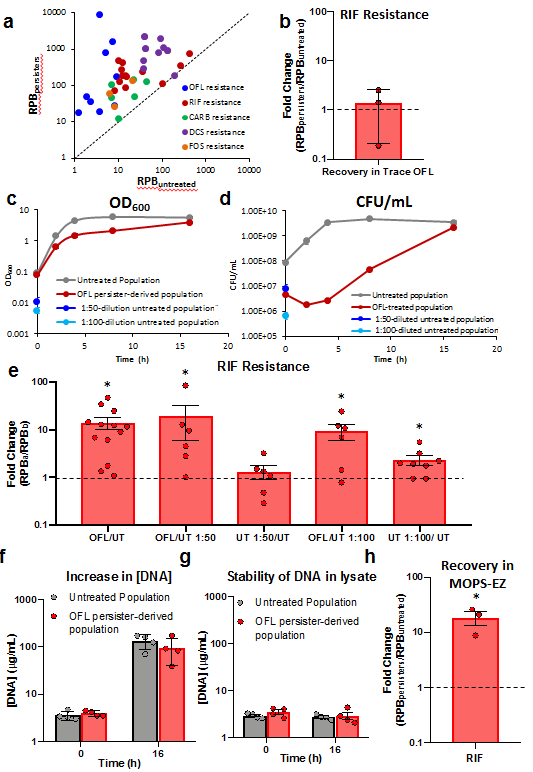


**Supplementary Fig. 6. Data and controls for resistance development assays. (a)** Scatterplot depicting resistant colonies per billion CFUs (RPB) from the OFL persister-derived population (RPB_persisters_) and untreated population (RPB_untreated_) measured in each replicate. The diagonal line depicts where the data points would lie if resistance enhance was not observed. Fold changes in Figure 3a were calculated from this data. At least three biological replicates were performed for each antibiotic examined. **(b)** The presence of trace amounts of OFL (0.024 ng/mL) in the recovery media did not lead to enhanced RIF resistance in the untreated population (n = 3). **(c)** OD_600_ and **(d)** colony forming units (CFU) of the untreated control and the OFL-treated population were measured immediately after inoculation into LB and at 2, 4, 8, and 16 h of recovery. As the number of culturable cells in the OFL-treated population was approximately 50-fold lower compared with the untreated population upon inoculation in LB and it further decreased at 2 h, we decreased the inoculum of the untreated sample 50- and 100-fold. We compared the number of RIF **(e)** resistant colonies in each culture following 16 h of recovery in LB. Decreasing the initial inoculum of the untreated culture increased RIF resistance by two-fold or less compared with the undiluted untreated population. This is significantly less than the 14±4-fold increase in RIF resistance observed with the OFL persister-derived population compared with the untreated, undiluted population (n = 13 for OFL/ UT; n = 6 for OFL/ UT 1:50, UT 1:50/ UT, and OFL/ UT 1:100; n = 8 for UT 1:100/ UT). **(f)** Increase in total DNA in the untreated and the OFL persister-derived populations during the 16 h recovery in LB was quantified using PicoGreen. Total DNA in the untreated population at 0 and 16 h were not statistically different from total DNA present in the OFL persister-derived population (n = 4). **(g)** DNA in cell lysates remained stable, and incubation of filtered cell lysate collected immediately after inoculation at 37 ^o^C overnight did not result in significant decreases in DNA (n = 4). **(h)** Recovery in MOPS-EZ media following OFL treatment resulted in significant increases in RIF resistant mutants (n = 3). Data are averages of three or more independent biological replicates. Error bars portray S.E.M., and * indicates significance (p-value ≤0.05) determined using two-tailed t-tests with unequal variances on log-transformed values and a value of 0 (log_10_ of a mean of 1) for panels **b**, **e**, and **h**.


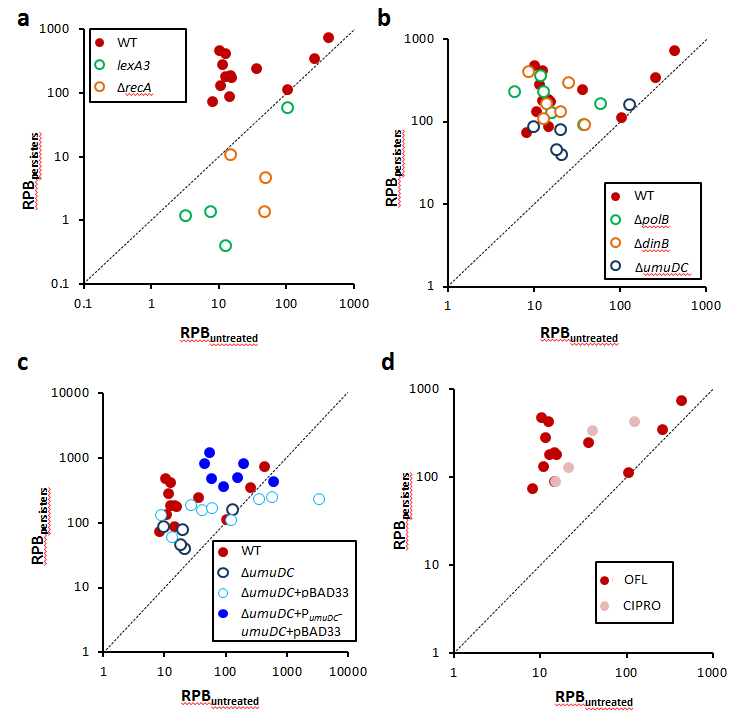


**Supplementary Fig. 7. Raw Data of RIF resistance enhancement in mutants.** Scatter plots depicting RIF resistant colonies per billion CFUs (RPB) from the OFL persister-derived population and untreated population measured in each replicate for **(a)** Δ*recA* and *lexA3* mutants, **(b)** mutants bearing deletions of each error-prone polymerase, and **(c)** Δ*umuDC* bearing pBAD33 or P_umuDC_-*umuDC* in pBAD33 (pTB02). The diagonal line depicts where the data points would lie if resistance enhancement was not observed. Fold changes in Figure 4 were calculated from this data. At least three biological replicates were performed for each mutant examined. **(d)** Scatter plot depicts RIF resistant colonies per billion CFUs (RPB) from the CIPRO persister-derived population and untreated population measured in each replicate. Fold changes in Figure 5d were calculated from this data. *indicates significance (p-value ≤0.05) determined using two-tailed t-tests with unequal variances on log-transformed values and 0 (log_10_ of a mean of 1).


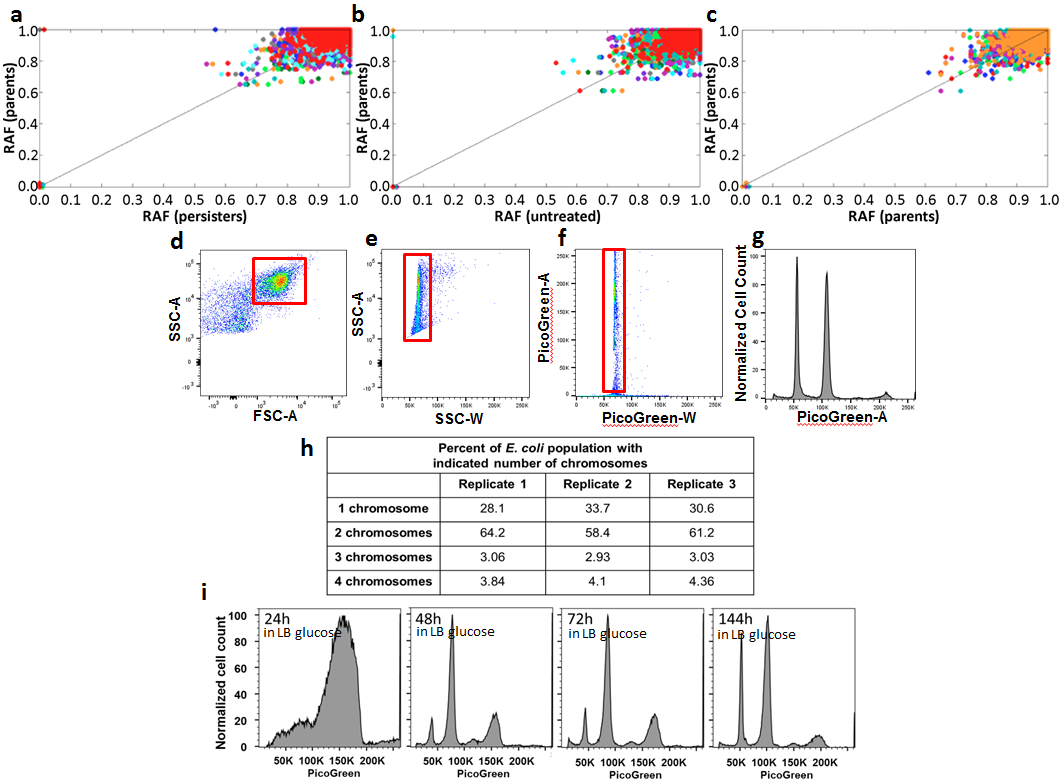


**Supplementary Fig. 8. Persisters repair DNA with high fidelity.** Reference allele frequencies (RAFs) of individual nucleotides in each of the parent populations were plotted against the RAF of the corresponding nucleotide in the colony derived from persisters **(a)** and untreated controls **(b)**. Parent v parent plots **(c)** are also provided for reference. Different colors represent different individual comparisons (*e.g.*, persister compared to its parent, untreated compared to its parent, parent 1 compared to parent 2). As depicted by the points at (0,0), there were two nucleotides that consistently demonstrated mutations in the parent colonies. The mutation in *glgP* is a C to T transition at nucleotide 3565749 in amino acid 278, changing a glycine to a glutamic acid. The mutation in *mdtO* is an A to T transversion at nucleotide 4302522 in amino acid 186, changing a methionine to a lysine. These mutations were also present in the progeny that arose from untreated cells and OFL persisters, and no new mutations consistently arose in progeny that were treated or untreated. **(d)** Gating strategy (red box) to visualize the total cell *E. coli* population using forward scatter (FSC) and side scatter (SSC), with a threshold set on side scatter. For doublet discrimination, cells were further analyzed and gated based on **(e)** side scatter area and width, and again via **(f)** PicoGreen fluorescence area and width. **(g)** PicoGreen fluorescence, corresponding to DNA content, was shown on a linear fluorescence scale. 50,000 PicoGreen single cell events were collected for each sample data file. 10,000 PicoGreen single events are shown in **(d-f). (h)** Chromosomal content of stationary-phase cultures prior to OFL treatment was determined using PicoGreen stains, and results from three biological replicates are summarized. **(i)** Fluorescence intensities corresponding to unit chromosomal copies were determined with PicoGreen staining of cells cultured to late stationary phase (Supplementary Methods). As culture time increased, more cells with a single chromosome emerged, indicating that a single chromosome corresponds to ~50,000 arbitrary fluorescence units.

**Supplementary Table 1. Mutations in rifampicin-resistant colonies.**

| **Mutations in RIF^R^ colonies** | | |
| --- | --- | --- |
|  | ***rpoB*** | **Outside *rpoB*** |
| **RIF^R^_parent_ 1** | *rpoB*C1537A | none |
| **RIF^R^_parent_ 2** | *rpoB*C1592T | *insF4*C764A  *insH10*G111T  *insA6*T88C |
| **RIF^R^_parent_ 3** | *rpoB*C1565T | *pncA*T452A  *insH7*G111T  *insA6*T88C |
| **RIF^R^_parent_ 4** | *rpoB*C1565T | *insH21*G111T  *pncA*T452A  *insA6*T88C |
| **RIF^R^_parent_ 5** | *rpoB*C1576T | *insF4*C764A  *dgcJ*TG989CC  *insH10*G111T |
| **RIF^R^_parent_ 6** | *rpoB*A1577T | *insH10*G111T |
| **RIF^R^_parent_ 7** | *rpoB*A1714T | *insA5*T88C  *insA6*T88C  *rhsA*G207C |
| **RIF^R^_parent_ 8** | *rpoB*A1714T | *insH10*G111T  *insA6*T88C |
| **RIF^R^_untreated_ 1** | *rpoB*A1714T | *rrlD*A1987G  *insA6*T88C |
| **RIF^R^_untreated_ 2** | *rpoB*A1547T | *insF4*C764A  *insA6*T88C |
| **RIF^R^_untreated_ 3** | none^a^ | *insH10*G111T  *insA6*T88C |
| **RIF^R^_untreated_ 4** | none^b^ | none^b^ |
| **RIF^R^_untreated_ 5** | *rpoB*A1538T | *insH7*G111T  *insH10*G111T  *insA6*T88C |
| **RIF^R^_untreated_ 6** | *rpoB*C1535A | *crl*A705G  *insF4*C764A  *rrlD*A1987G  *insA6*T88C |
| **RIF^R^_untreated_ 7** | *rpoB*C1576T | *insF4*C764A  *insH10*G111T  *insA6*T88C |
| **RIF^R^_untreated_ 8** | *rpoB*C1576T | *insH10*G111T  *rrlD*A1987G  *insA6*T88C |
| **RIF^R^_OFL persister_ 1** | *rpoB*C1576A | *insH10*G111T  *rrlD*A1987G |
| **RIF^R^_OFL persister_ 2** | *rpoB*C1576A | *insA6*T88C |
| **RIF^R^_OFL persister_ 3** | *rpoB*C1576A | *insF4*C764A  *insH10*G111T  *insA6*T88C |
| **RIF^R^_OFL persister_ 4** | *rpoB*C1592A | *insH10*G111T  *insA6*T88C |
| **RIF^R^_OFL persister_ 5** | *rpoB*C1576G | *insH10*G111T  *insA6*T88C |
| **RIF^R^_OFL persister_ 6** | *rpoB*A1538T | *insI1*A548C  *insF4*C764A  *ansA*G472A  *insH8*G111T  *insH10*G111T  *insA6*T88C |
| **RIF^R^_OFL persister_ 7** | *rpoB*A1538T | *insB4*T239G  *insH10*G111T  *insA6*T88C |
| **RIF^R^_OFL persister_ 8** | *rpoB*C1576G | *dgcJ*G990C  *insH10*G111T  *insA6*T88C  *rhsB*G207C |

**RIF^R^_parent_** indicates RIF^R^ colony from parent population prior to treatment

**RIF^R^_untreated_** indicates RIF^R^ colony from untreated population (water in place of OFL)

**RIF^R^_OFL persister_** indicates RIF^R^ colony from OFL persister population

^a^**RIF^R^_untreated_** 3 bore *rpoB*C1565T in 42% of reads, which was below the 50% cutoff

^b^**RIF^R^_untreated_** 4 did not bear any mutations within the sequencing data other than those that are known to be present in the wild-type background. For that colony, we hypothesize that mutations conferring RIF resistance may occur at a locus that was not sequenced at a sufficient depth for analysis.

*rpoB* mutations observed here have been reported in RIF-resistant *E. coli* and in homologous regions in clinical isolates^24, 25^.

All colonies had three mutations found to be in the MG1655 population used for this work: *glgP*G833A, *mdtO*T557A, and a GC insertion following nucleotide 11 in repetitive element REP321j.

**Supplementary Table 2. Bacterial strains and plasmids.**

| **Strain** | **Genotype** | **Source or Reference** |
| --- | --- | --- |
| MG1655 | F^-^ λ^-^ *ilvG*^-^ *rfb*-50 *rph*-1 | ATCC 700926 ^22^ |
| TB001 | MG1655 Δ*recA* | This work, generated by deletion of *recA* using the Datsenko-Wanner method, cured of KAN^R^ by pCP20 ^1^ |
| TB002 | MG1655 Δ*malK*::KAN^R^ | Generated by P1 phage transduction of Δ*malK::*KAN^R 3^ into MG1655 |
| TB003 | MG1655 *lexA3* | Generated by P1 phage transduction of *lexA3* ^3^ into TB002 |
| TB004 | MG1655 Δ*polB* | Generated by P1 phage transduction of Δ*polB::*KAN^R 2^ into MG1655, cured of KAN^R^ by pCP20 ^1^ |
| TB005 | MG1655 Δ*dinB* | Generated by P1 phage transduction of Δ*dinB::*KAN^R 2^ into MG1655, cured of KAN^R^ by pCP20 ^1^ |
| TB006 | MG1655 Δ*umuDC* | Generated by deletion of *umuDC* using the Datsenko-Wanner method, cured of KAN^R^ by pCP20 ^1^ |
| TB007 | MG1655 Δ*ruvA* | Generated by P1 phage transduction of Δ*ruvA::*KAN^R 2^ into MG1655, cured of KAN^R^ by pCP20 ^1^ |
| TB008 | MG1655 Δ*recG* | Generated by P1 phage transduction of Δ*recG::*KAN^R 2^ into MG1655, cured of KAN^R^ by pCP20 ^1^ |
| TB009 | MG1655 Δ*recB* | Generated by P1 phage transduction of Δ*recB::*KAN^R 2^ into MG1655, cured of KAN^R^ by pCP20 ^1^ |
| TB010 | MG1655 Δ*ruvA* Δ*recG* | Generated by P1 phage transduction of Δ*recG::*KAN^R 2^ into TB007*,* cured of KAN^R^ by pCP20 ^1^ |
| **Plasmid** | | **Source or Reference** |
| pUA66 | Vector, SC101 ori, KAN^R^, *gfpmut2* reporter | ^23^ |
| pBAD33 | Vector, p15A ori, CM^R^ | ^5^ |
| pKV006 | pKV000 P*_recA_-gfpmut2*, KAN^R^ | ^3^ |
| pTB01 | pBAD33 P*_recA_-recA*, CM^R^ | This work |
| pTB02 | pBAD33 P*_umuDC_*-*umuDC*, CM^R^ | This work |

**Supplementary Table 3. Oligonucleotides for mutant and plasmid construction and confirmation**

| **Oligonucleotides for mutant construction** | | | | | |
| --- | --- | --- | --- | --- | --- |
| **Mutation** | **Forward (5'🡪3')** | | | **Reverse (5'🡪3')** | |
| Δ*recA*::KAN^R^ *^a^* | CAACAGAACATATTGACTATCCGGTATTACCCGGCATGACAGGAGTAAAAATGGTGTAGGCTGGAGCTGCTTCG | | | ATGCGACCCTTGTGTATCAAACAAGACGATTAAAAATCTTCGTTAGTTTCCATATGAATATCCTCCTTAGTTCCTATTC | |
| Δ*umuDC*::KAN^R^ | CTACTGTATATAAAAACAGTATAACTTCAGGCAGATTATTGTGTAGGCTGGAGCTGCTTC | | | TCGGCGCTCCTGCGGGAGCGCTTTTTTCCTGCCGCTATATCATATGAATATCCTCCTTAG | |
| **Oligonucleotides for plasmid construction (Gibson Assembly)** | | | | | |
| **Plasmid/Insert** | **Forward (5'🡪3')** | | | **Reverse (5'🡪3')** | |
| pBAD33 | CCTGCAGGCATGCAAGCTTG | | | GGTACCGATAAGCTGTCAAACATGAGC | |
| P*_recA_-recA*  (pTB01) | tttgacagcttatcggtaccAAAACACTTGATACTGTATGAGC | | | caagcttgcatgcctgcaggTTAAAAATCTTCGTTAGTTTCTG | |
| P*_umuDC_-umuDC* (pTB02) | tttgacagcttatcggtaccAATCAGTATTGATCTGCTGG | | | GCTCATGTTTGACAGCTTATCTTATTTGACCCTCAGTAAATCAG | |
| **Oligonucleotides to confirm genetic mutants** | | | | | |
| **Mutation** | | **Upstream Forward**  **Primer**  **(5'🡪3')** | **Downstream Reverse**  **Primer**  **(5'🡪3')** | **Internal Forward Primer**  **(5'🡪3')** | **Internal Reverse**  **Primer**  **(5'🡪3')** |
| Δ*recA*::KAN^R^  Δ*recA* | | CTGGTTTGCTTTTGCCACTG | AATACGCGCAGGTCCATAAC | TGGAAACCATCTCTACCGGTTC | GACGAACAGAGGCGTAGAAT |
| Δ*malK*::KAN^R^ | | GCCAGGGGGTGGAGGATTTAAGC |  | CCGTCTGGCTGCGGTAAATC | TGCCATCCTCACGGAACAGA |
| *lexA3* | | TTCCAAAATCGCCTTTTGCT |  | GTTAACGGCCAGGCAACAAG | GCCCTTCAATGGTGAAGCTCT |
| Δ*polB*::KAN^R^  Δ*polB* | | AAGGCATATTACGGGCAGTA | TGGTGTTATTCAGGTCCAGG | AGCCTGATCCAGAGCACAGT | TCAGCCAGACAAACGTTGAG |
| Δ*dinB*::KAN^R^  Δ*dinB* | | CCGATACGCTGTATCAATACTTTG | ATACCCGCATCCTTATTCCTT | GTGATCAGCACCGCCAATTA | GGTCAAGCAACGTCACATGC |
| Δ*umuDC*::KAN^R^  Δ*umuDC* | | GCCTATGCAGCGACAAATAT | GGTGGTGATTTCCCAGCCGT | TTTATCAAGCCTGCGGATCT | TGCCAAAATCAGTCAGATCG |
| Δ*ruvA*::KAN^R^  Δ*ruvA* | | CATCGAGACACCTCGCAAGTT | AACAAATGATCGAGGGCATC | AAGTGGGCGGCGTAGGCTAT | GCGGCTTGCTTCTTGTGGTT |
| Δ*recG*::KAN^R^  Δ*recG* | | CAAAACGCAAAGGCCTGCCT | TATCGTCAATGCGCTGGTAA | GGTTTGCACCGCTCGGTATC | CCGGCATCCAGCGTTCTATC |
| Δ*recB*::KAN^R^  Δ*recB* | | AACGGGAAAGCCGAATATGTACAC | ATCAAGATGCAGCGGGTTAC | GCGGAAGATCTGCGTTTGCT | TCATAGCGGTGTGCCTGCAT |

Internal KAN^R^ cassette reverse primer for confirmation of genetic mutants (5'🡪3'): ATGATGGATACTTTCTCGGCAGGAG

***^a^***Primers consist of approximately 50 nucleotide (nt) homology regions for target locations in chromosome and 20-30 nt sequence to amplify KAN^R^ cassette from pKD4^1^.

**References for Supplementary Information**

1. Datsenko, K. A. & Wanner, B. L. One-step inactivation of chromosomal genes in *Escherichia coli* K-12 using PCR products. *Proc. Natl. Acad. Sci. U. S. A.* **97**, 6640-6645, doi:10.1073/pnas.120163297 (2000).

2. Baba, T.*et al.* Construction of *Escherichia coli* K-12 in-frame, single-gene knockout mutants: the Keio collection. *Mol. Syst. Biol.* **2**, 2006.0008, doi:10.1038/msb4100050 (2006).

3. Völzing, K. G. & Brynildsen, M. P. Stationary-Phase Persisters to Ofloxacin Sustain DNA Damage and Require Repair Systems Only during Recovery. *mBio* **6**, e00731-00715, doi:10.1128/mBio.00731-15 (2015).

4. Gibson, D. G. *et al.* Enzymatic assembly of DNA molecules up to several hundred kilobases. *Nature Methods* **6**, 343-345, doi:10.1038/nmeth.1318 (2009).

5. Guzman, L. M., Belin, D., Carson, M. J. & Beckwith, J. Tight regulation, modulation, and high-level expression by vectors containing the arabinose PBAD promoter. *J. Bacteriol.* **177**, 4121-4130 (1995).

6. Keren, I., Kaldalu, N., Spoering, A., Wang, Y. & Lewis, K. Persister cells and tolerance to antimicrobials. *FEMS Microbiol. Lett.* **230**, 13-18 (2004).

7. Leung, V. & Lévesque, C. M. A stress-inducible quorum-sensing peptide mediates the formation of persister cells with noninherited multidrug tolerance. *J. Bacteriol.* **194**, 2265-2274, doi:10.1128/JB.06707-11 (2012).

8. Young, J. *et al.* Measuring single-cell gene expression dynamics in bacteria using fluorescence time-lapse microscopy. *Nature Protoc.* **7**, 80-88, doi:10.1038/nprot.2011.432 (2011).

9. Preibisch, S., Saalfeld, S. & Tomancak, P. Globally optimal stitching of tiled 3D microscopic image acquisitions. *Bioinformatics* **25**, 1463-1465, doi:10.1093/bioinformatics/btp184 (2009).

10. Schindelin, J. *et al.* Fiji: an open-source platform for biological-image analysis. *Nature Methods* **9**, 676-682, doi:10.1038/nmeth.2019 (2012).

11. Rueden, C. T. *et al.* ImageJ2: ImageJ for the next generation of scientific image data. *BMC Bioinformatics* **18**, 529, doi:10.1186/s12859-017-1934-z (2017).

12. Ducret, A., Quardokus, E. M. & Brun, Y. V. MicrobeJ, a tool for high throughput bacterial cell detection and quantitative analysis. *Nature Microbiol.* **1**, 16077, doi:10.1038/nmicrobiol.2016.77 (2016).

13. Blankenberg, D. *et al.* Galaxy: a web-based genome analysis tool for experimentalists. *Curr. Protoc. Mol. Biol.* **Chapter 19**, Unit 19.10.1-21, doi:10.1002/0471142727.mb1910s89 (2010).

14. Giardine, B. *et al.* Galaxy: a platform for interactive large-scale genome analysis. *Genome Res.* **15**, 1451-1455, doi:10.1101/gr.4086505 (2005).

15. Goecks, J., Nekrutenko, A., Taylor, J. & Galaxy Team. Galaxy: a comprehensive approach for supporting accessible, reproducible, and transparent computational research in the life sciences. *Genome Biol.* **11**, R86, doi:10.1186/gb-2010-11-8-r86 (2010).

16. Li, H. & Durbin, R. Fast and accurate short read alignment with Burrows-Wheeler transform. *Bioinformatics* **25**, 1754-1760, doi:10.1093/bioinformatics/btp324 (2009).

17. Li, H. & Durbin, R. Fast and accurate long-read alignment with Burrows-Wheeler transform. *Bioinformatics* **26**, 589-595, doi:10.1093/bioinformatics/btp698 (2010).

18. Garrison, E. & Marth, G. Haplotype-based variant detection from short-read sequencing. Cornell University Library, arXiv:1207.3907 (2012).

19. Akerlund, T., Nordström, K. & Bernander, R. Analysis of cell size and DNA content in exponentially growing and stationary-phase batch cultures of *Escherichia coli*. *J. Bacteriol.* **177**, 6791-6797 (1995).

20. Ferullo, D. J., Cooper, D. L., Moore, H. R. & Lovett, S. T. Cell cycle synchronization of *Escherichia coli* using the stringent response, with fluorescence labeling assays for DNA content and replication. *Methods* **48**, 8-13, doi:10.1016/j.ymeth.2009.02.010 (2009).

21. Nielsen, H.J., Li, Y., Youngren, B., Hansen, F.G., & Austin, S. Progressive segregation of the *Escherichia coli* chromosome. *Mol. Microbiol.* **61,** 383-393, doi: 10.1111/j.1365-2958.2006.05245.x (2006).

22. Kohanski, M. A., Dwyer, D. J., Hayete, B., Lawrence, C. A. & Collins, J. J. A common mechanism of cellular death induced by bactericidal antibiotics. *Cell* **130**, 797-810, doi:10.1016/j.cell.2007.06.049 (2007).

23. Zaslaver, A.*et al.* A comprehensive library of fluorescent transcriptional reporters for *Escherichia coli*. *Nature Methods* **3**, 623-628, doi:10.1038/nmeth895 (2006).

24. Ramaswamy, S. & Musser, J. M. Molecular genetic basis of antimicrobial agent resistance in *Mycobacterium tuberculosis*: 1998 update. *Tuber. Lung Dis.* **79**, 3-29, doi:10.1054/tuld.1998.0002 (1998).

25. Wolff, E., Kim, M., Hu, K., Yang, H. & Miller, J. H. Polymerases leave fingerprints: analysis of the mutational spectrum in *Escherichia coli rpoB* to assess the role of polymerase IV in spontaneous mutation. *J. Bacteriol.* **186**, 2900-2905 (2004).
