# Supplementary material for: Enhanced antibiotic resistance development from fluoroquinolone persisters after a single exposure to antibiotic
Source: Nat Commun. 2019 Mar 12;10:1177. doi: 10.1038/s41467-019-09058-4 (PMC6414640; doi:10.1038/s41467-019-09058-4)
Supplement: Supplementary file 3 — Description of Additional Supplementary Files [file 41467_2019_9058_MOESM3_ESM.docx]

**Description of Supplementary Files**

**File Name:** Supplementary Movie 1.

**Description:** Time-lapse microscopy of recovering OFL-treated cells - Replicate 1. Phase contrast and GFP fluorescence images were taken every 12 min as OFL-treated *E. coli* MG1655 carrying a P*_recA_*-gfp reporter plasmid recovered from treatment. This movie is representative of six biological replicates. Images from Figure 1a were captured from frames from this movie.

**File Name:** Supplementary Movie 2

**Description:** Time-lapse microscopy of recovering OFL-treated cells - Replicate 2. Phase contrast and GFP fluorescence images were taken every 12 min as OFL-treated *E. coli* MG1655 carrying a P*_recA_*-gfp reporter plasmid recovered from treatment. This movie is representative of six biological replicates.

**File Name:** Supplementary Movie 3

**Description:** Time-lapse microscopy of untreated control. Phase contrast and GFP fluorescence images were taken every 12 min as an untreated control of *E. coli* MG1655 carrying a P*_recA_*-gfp reporter plasmid recovered. This movie is representative of six biological replicates. Images from Supplementary Figure 3a were captured from frames from this movie.

**File Name:** Supplementary Movie 4.

**Description:** Time-lapse microscopy of untreated control recovered on trace OFL. Phase contrast and GFP fluorescence images were taken every 12 min as an untreated control of *E. coli* recovered on ~0.2 ng/mL OFL. The presence of trace OFL did not induce the SOS response in the untreated population. This movie is representative of two biological replicates. The cell lengths and fluorescence of 15 of these cells were quantified in Supplementary Figure 3c.

**File Name:** Supplementary Movie 5

**Description:** Time-lapse microscopy of recovering OFL-treated MG1655 Δ*recA* mutant. Phase contrast and GFP fluorescence images were taken every 12 min as OFL-treated *E. coli* MG1655 Δ*recA* carrying a P*_recA_*-gfp reporter plasmid recovered from treatment. This movie is representative of two biological replicates. Images from Figure 2 were captured from frames from this video. We did not observe considerable filamentation or SOS induction following OFL treatment in the *recA* mutant, and we did not observe any persisters.

**File Name:** Supplementary Movie 6

**Description:** Time-lapse microscopy of untreated MG1655 Δ*recA* mutant. Phase contrast and GFP fluorescence images were taken every 12 min as untreated *E. coli* MG1655 Δ*recA* carrying a P*_recA_*-gfp reporter plasmid recovered. This movie is representative of two biological replicates. Images from Figure 2 were captured from frames from this video.

**File Name:** Supplementary Movie 7

**Description:** Time-lapse microscopy of recovering OFL-treated MG1655 *lexA3* mutant. Phase contrast and GFP fluorescence images were taken every 12 min as OFL-treated *E. coli* MG1655 *lexA3* carrying a P*_recA_*-gfp reporter plasmid recovered from treatment. This movie is representative of two biological replicates. Images from Figure 2 were captured from frames from this video. We did not observe considerable filamentation or SOS induction following OFL treatment in *lexA3* mutants, and we did not observe any persisters.

**File Name:** Supplementary Movie 8

**Description:** Time-lapse microscopy of untreated MG1655 *lexA3* mutant. Phase contrast and GFP fluorescence images were taken every 12 min as untreated *E. coli* MG1655 *lexA3* carrying a P*_recA_*-gfp reporter plasmid recovered. This movie is representative of two biological replicates. Images from Figure 2 were captured from frames from this video.
