# Supplementary material for: Enhanced antibiotic resistance development from fluoroquinolone persisters after a single exposure to antibiotic
Source: Nat Commun. 2019 Mar 12;10:1177. doi: 10.1038/s41467-019-09058-4 (PMC6414640; doi:10.1038/s41467-019-09058-4)
Supplement: Supplementary file 12 — Reporting Summary [file 41467_2019_9058_MOESM12_ESM.pdf]

nature research

Corresponding author(s): Mark P. Brynildsen

Last updated by author(s): Feb 4, 2019

Reporting Summary

Nature Research wishes to improve the reproducibility of the work that we publish. This form provides structure for consistency and transparency in reporting. For further information on Nature Research policies, see Authors & Referees and the Editorial Policy Checklist.

Statistics

For all statistical analyses, confirm that the following items are present in the figure legend, table legend, main text, or Methods section.

n/a	Confirmed	
☐	☒	The exact sample size (*n*) for each experimental group/condition, given as a discrete number and unit of measurement
☐	☒	A statement on whether measurements were taken from distinct samples or whether the same sample was measured repeatedly
☐	☒	The statistical test(s) used AND whether they are one- or two-sided<br>*Only common tests should be described solely by name; describe more complex techniques in the Methods section.*
☐	☒	A description of all covariates tested
☐	☒	A description of any assumptions or corrections, such as tests of normality and adjustment for multiple comparisons
☐	☒	A full description of the statistical parameters including central tendency (e.g. means) or other basic estimates (e.g. regression coefficient) AND variation (e.g. standard deviation) or associated estimates of uncertainty (e.g. confidence intervals)
☐	☒	For null hypothesis testing, the test statistic (e.g. *F*, *t*, *r*) with confidence intervals, effect sizes, degrees of freedom and *P* value noted<br>*Give P values as exact values whenever suitable.*
☒	☐	For Bayesian analysis, information on the choice of priors and Markov chain Monte Carlo settings
☒	☐	For hierarchical and complex designs, identification of the appropriate level for tests and full reporting of outcomes
☒	☐	Estimates of effect sizes (e.g. Cohen's *d*, Pearson's *r*), indicating how they were calculated

Our web collection on statistics for biologists contains articles on many of the points above.

Software and code

Policy information about availability of computer code

Data collection	MicrobeJ [Ducret A, Quardokus EM, and Brun YV. MicrobeJ, a tool for high throughput bacterial cell detection and quantitative analysis. Nat. Microbiol. 1, 16077 (2016)] was used to quantify growth and fluorescence of cells in time-lapse microscopy images. The versions available between August 2015 and November 2018 were used for analysis.
Data analysis	Galaxy [Giardine B et. al. Galaxy: a platform form interactive large-scale genome analysis. Genome Res. 15:1451-1455 (2005)] was used to analyze whole-genome sequencing data. Within Galaxy, the Burrows-Wheeler Alignment tool [Li H and Durbin R. Fast and accurate short read alignment with Burrows-Wheeler transform. Bioinformatics. 25, 1754-1760 (2009)] was used to map reads to the reference chromosome and FreeBayes [Garrison E and Marth G. Haplotype-based variant detection from short-read sequencing. arXiv preprint arXiv:1207.3907 [q-bio.GN] (2012)] was used to detect variants from the reference chromosome. The versions available between July 2016 and June 2017 were used.

For manuscripts utilizing custom algorithms or software that are central to the research but not yet described in published literature, software must be made available to editors/reviewers. We strongly encourage code deposition in a community repository (e.g. GitHub). See the Nature Research guidelines for submitting code & software for further information.

Data

Policy information about availability of data

All manuscripts must include a data availability statement. This statement should provide the following information, where applicable:

- Accession codes, unique identifiers, or web links for publicly available datasets
- A list of figures that have associated raw data
- A description of any restrictions on data availability

Sequencing data that support the findings of this study have been deposited in the BioProject Database with the accession code of PRJNA517575. Additional data that support the findings of this study are available from the corresponding author upon reasonable request.

Field-specific reporting

Please select the one below that is the best fit for your research. If you are not sure, read the appropriate sections before making your selection.

☒ Life sciences ☐ Behavioural & social sciences ☐ Ecological, evolutionary & environmental sciences

For a reference copy of the document with all sections, see nature.com/documents/nr-reporting-summary-flat.pdf

Life sciences study design

All studies must disclose on these points even when the disclosure is negative.

Sample size	Sample size calculations were not performed. All experiments were performed three times (biological replicates), and additional replicates were performed when variability was considered high compared to the mean.
Data exclusions	Data was not excluded; all data collected from the specified experiments were reported and used in analyses.
Replication	All experiments were performed at a minimum in biological triplicate. Experiments were performed by three separate authors independently, and data for several assays were collected by two or more independent authors and combined for analysis.
Randomization	This is not relevant because all data reported here were from Escherichia coli MG1655 and its derivatives.
Blinding	Investigators were not blinded to sample identity during data collection or analysis. The metrics used in this study were not subjective, and blinding investigators to sample identity is not standard practice in microbiology.

Reporting for specific materials, systems and methods

We require information from authors about some types of materials, experimental systems and methods used in many studies. Here, indicate whether each material, system or method listed is relevant to your study. If you are not sure if a list item applies to your research, read the appropriate section before selecting a response.

Materials & experimental systems

n/a	Involved in the study
☒	☐ Antibodies
☒	☐ Eukaryotic cell lines
☒	☐ Palaeontology
☒	☐ Animals and other organisms
☒	☐ Human research participants
☒	☐ Clinical data

Methods

n/a	Involved in the study
☒	☐ ChIP-seq
☐	☒ Flow cytometry
☒	☐ MRI-based neuroimaging

Flow Cytometry

Plots

Confirm that:

☒ The axis labels state the marker and fluorochrome used (e.g. CD4-FITC).

☒ The axis scales are clearly visible. Include numbers along axes only for bottom left plot of group (a 'group' is an analysis of identical markers).

☒ All plots are contour plots with outliers or pseudocolor plots.

☒ A numerical value for number of cells or percentage (with statistics) is provided.

Methodology

Sample preparation	Stationary phase E. coli were fixed in 70% ethanol and stained with PicoGreen.
Instrument	LSRII flow cytometer (BD Biosciences, San Jose, CA)
Software	FlowJo Software Version 9.8 (TreeStar, Ashland, OR)
Cell population abundance	Sorting was not conducted.
Gating strategy	The total cell E. coli population was visualized using forward scatter and side scatter (log scale for both), with a threshold set on side scatter. For doublet discrimination, cells were further analyzed and gated based on side scatter area and width, and again

via PicoGreen fluorescence area and width. PicoGreen fluorescence, corresponding to DNA content, was shown on a linear fluorescence scale. 50,000 PicoGreen single cell events were collected for each sample data file.

☒ Tick this box to confirm that a figure exemplifying the gating strategy is provided in the Supplementary Information.
